# The pleiotropic roles of autophagy in Alzheimer's disease: From pathophysiology to therapy

**DOI:** 10.1016/j.coph.2021.07.011

**Published:** 2021-10

**Authors:** Beatrice Paola Festa, Antonio Daniel Barbosa, Matea Rob, David C. Rubinsztein

**Affiliations:** 1 Department of Medical Genetics, Cambridge Institute for Medical Research (CIMR), University of Cambridge, Cambridge, UK; 2 UK Dementia Research Institute, Cambridge Institute for Medical Research (CIMR), University of Cambridge, Cambridge, UK

## Abstract

Autophagy is a lysosomal degradation pathway and the main clearance route of many toxic protein aggregates. The molecular pathology of Alzheimer's disease (AD) manifests in the form of protein aggregates—extracellular amyloid-β depositions and intracellular tau neurofibrillary tangles. Perturbations at different steps of the autophagy pathway observed in cellular and animal models of AD might contribute to amyloid-β and tau accumulation. Increased levels of autophagosomes detected in patients' brains suggest an alteration of autophagy in human disease. Autophagy is also involved in the fine-tuning of inflammation, which increases in the early stages of AD and possibly drives its pathogenesis. Mounting evidence of a causal link between impaired autophagy and AD pathology uncovers an exciting opportunity for the development of autophagy-based therapeutics.

## Abbreviations

5-HT2AR: 5-Hydroxytryptamine receptor 2A
AKT (PKB): Protein kinase B
AMPK: AMP-activated protein kinase
ANP32A: Acidic leucine-rich nuclear phosphoprotein 32 family member A
APP: Amyloid precursor protein
ATG: Autophagy-related gene
ATP: Adenosine triphosphate
BECN1: Beclin 1
CBP: CREB-binding protein
cIAP1/2: Cellular inhibitor of apoptosis protein 1/2
CREB: cAMP response element-binding protein
Erβ: Oestrogen receptor beta
GSK3β: Glycogen synthase kinase 3 beta
H3R: Histamine 3 receptor
HDAC6: Histone deacetylase 6
IFN-γ: Interferon gamma
IL: Interleukin
INHAT: Inhibitor of acetyltransferases
IST1: Increased sodium tolerance protein 1
LAMP1: Lysosomal-associated membrane protein 1
LC3: Microtubule-associated proteins 1A/1B light chain 3B
mGluR5: Metabotropic glutamate receptor 5
mTOR: Mammalian target of rapamycin
mTORC1: Mammalian target of rapamycin complex 1
NBRF2: Neighbour of BRCA1 LncRNA 2
NLRP3: NOD-, LRR- and pyrin domain-containing protein 3
OPTN: Optineurin
p300: Histone acetyltransferase p300
p62/SQSTM1: Sequestosome-1
PI3P: Phosphatidylinositol 3-phosphate
PPARα: Peroxisome proliferator-activated receptor alpha
PrPc: Cellular prion protein
PSEN1/2: Presenilin 1/2
RAB7: RAS-related protein Rab-7a
RAPTOR: Regulatory associated protein of mTOR
RIPK1: Receptor-interacting serine/threonine-protein kinase 1
RPS6KB1: Ribosomal protein S6 kinase beta-1
SIRT1: Sirtuin-1
SNAPIN: SNARE-associated protein
SNARE: SNAP receptor
SSH1: Slingshot homologue 1
TFEB: Transcription factor EB
TNF: Tumour necrosis factor
TRADD: TNF receptor type-1 associated death domain
TRAF2: TNF Receptor-associated factor 2
ULK1/2: Unc-51 like autophagy activating kinase
VDAC1: Voltage-dependent anion-selective channel 1
VPS34: Vacuolar protein sorting 34
WIPI2: WD repeat domain phosphoinositide-interacting 2
ZBTB16: Zinc finger and BTB domain-containing protein 16

## Autophagy

Macroautophagy (hereafter referred to as autophagy) is an evolutionarily conserved pathway where portions of cytoplasm are sequestered in double-membraned autophagosomes and delivered to lysosomes for degradation. Autophagy allows recycling of nutrients to provide energy during starvation, but in complex multicellular organisms, it is also involved in other aspects of cellular stress management. Indeed, by degrading aggregate-prone proteins and damaged organelles, autophagy acts as aquality control machinery maintaining cellular homeostasis [1]. Furthermore, autophagy negatively regulates several key components of the immune response, playing a crucial role in counteracting inflammation [2]. Autophagy is particularly important in safeguarding the health of brain cells, and its impairment results in the neuronal accumulation of toxic proteins and excessive neuroinflammation—hallmarks of many neurodegenerative diseases [3]. Although disrupted autophagy may not be the primary cause of neurodegeneration, a growing body of evidence indicates that defects at different stages of this pathway may contribute to disease progression and pathology, suggesting that boosting autophagy might be a valuable therapeutic strategy.

**Figure 1:**
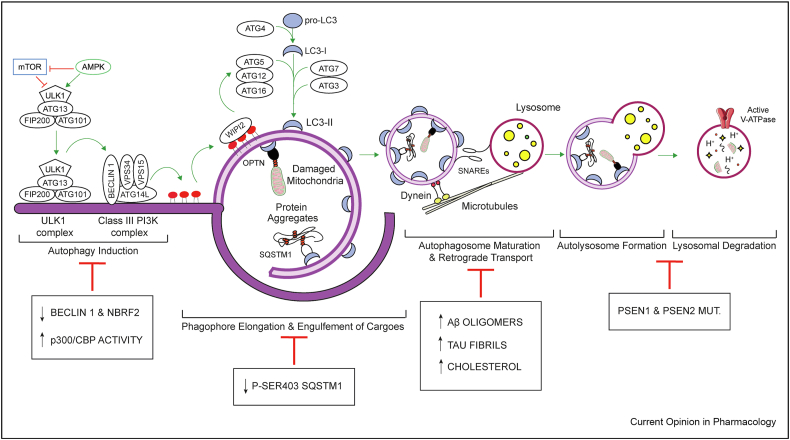
**Autophagy in the pathogenesis of AD.** mTOR inhibition and AMPK activation are the most common signalling pathways inducing autophagy. Both events lead to the activation of the ULK1 complex (ULK1, ATG13, FIP200, ATG101), which, in turn, translocates to the pre-autophagosomal membrane where it starts the nucleation of the phagophore with the recruitment of the class III PI3K complex (BECLIN 1, VPS34, VPS15, ATG14L) and the production of PI3P. The latter interacts with WIPI2, which subsequently recruits the ATG5–ATG12–ATG16 complex (E3-like ligase). The coordinated action of this complex along with ATG7 (E1-like ligase) and ATG3 (E2-like ligase) allows the conjugation of LC3, previously cleaved by ATG4, to phosphatidylethanolamine (PE) lipids on the developing autophagosome. During the expansion, cytoplasmic materials (protein aggregates and damaged mitochondria) are engulfed in the phagophore through the interaction with specific adaptors (e.g. SQSTM1 and OPTN) directly binding to LC3-II. After closure, the newly formed autophagosome travels along microtubules, via dynein motor protein, to reach the lysosome. Autophagosome–lysosome fusion is aided by SNAREs and leads to the formation of the autolysosome. Proper acidification of lysosomes, mediated by v-ATPase, is necessary to activate the lysosomal hydrolases and stimulate proteolysis of the cytoplasmic materials. The white boxes describe the molecular events, which hinder the progression of the autophagy flux during the pathogenesis of AD.

Autophagy is a multistep process that starts with the formation of a cup-shaped, double-membraned structure called a phagophore. On activation by Unc-51 like autophagy activating kinase (ULK1/2), the vacuolar protein sorting 34 (VPS34)/Beclin1 complex translocates on the nascent phagophore to produce phosphatidylinositol 3-phosphate, which enables the sequential recruitment of WD repeat domain phosphoinositide-interacting 2 and the autophagy-related gene (ATG)12–ATG5–ATG16L1 complex. The latter is an E3-like ligase protein complex required for the conjugation of Atg8/microtubule-associated proteins 1A/1B light chain 3B (LC3) on the developing autophagosome. In addition to enabling expansion and closure of the phagophore, LC3 facilitates the engulfment of cargo in autophagic vesicles by directly interacting with autophagy receptors, such as sequestosome-1 (p62/SQSTM1). After closure, autophagosomes travel towards lysosomes clustered at the microtubule organising centre via dynein motors, where SNAP receptors (SNAREs) mediate autophagosome–lysosome fusion, enabling degradation of the autophagic cargo (Figure 1). Autophagy is triggered by various stressor-signalling pathways. The most common are low energy production–mediated AMP-activated protein kinase (AMPK) activation and nutrient starvation–mediated mammalian target of rapamycin complex 1 (mTORC1) inhibition. Transcription factor EB (TFEB), a transcription factor controlled by mTORC1, coordinates both autophagosome biogenesis and lysosomal proteolysis, acting as a central regulator of the autophagy flux [4].

In this review, we will consider how proteins linked to Alzheimer's disease (AD) affect autophagy and how autophagy disruption impacts the accumulation of the pathological amyloid-β (Aβ) and tau aggregates. Finally, we will discuss the most recent developments in autophagy-related therapeutics.

## Alzheimer's disease

AD is a debilitating neurodegenerative condition clinically characterised by progressive dementia and cognitive impairment. Central pathologic events in AD are as follows: (i) the abnormal cleavage of amyloid precursor protein (APP), resulting in the accumulation of Aβ plaques in the extracellular space, and (ii) the hyperphosphorylation of microtubule-associated protein tau, leading to the formation of intracellular neurofibrillary tangles (NFTs). Converging evidence indicates that the deposition of Aβ is a likely initial pathological event in AD and is responsible for the spread of tau pathology. Clinically, the amount of tau NFTs correlates with the cognitive dysfunction of AD to a greater extent than Aβ plaque load does. Chronic neuroinflammation—recently identified as a third core feature of AD—has emerged as a potential bridge between these two events [5]. Although most patients present with ‘sporadic’ late onset disease (~80 years), a small number of cases (<1%) have inherited mutations in genes that affect processing of Aβ (*APP*, *PSEN1* and *PSEN2*) and develop the disease much earlier (~45 years) [6]. The discovery of monogenic forms of AD has led to the development of faithful cellular and animal models of these Mendelian diseases, allowing a better investigation of the biological mechanisms underlying this condition, including autophagy.

## Autophagy and Alzheimer's disease

Perturbations at different stages of autophagy might compromise the homeostasis of toxic aggregate-prone proteins (Aβ and tau) and exacerbate neuroinflammation, thus contributing to AD pathogenesis. Supporting a relationship between autophagy and AD, genetic variants relevant to the autophagy-lysosomal pathway have been identified as disease risk factors for late-onset AD [6], and conversely, pathogenic aggregate-prone toxic proteins can also affect autophagy (Figure 1). The accumulation of immature autophagic vacuoles observed in dystrophic neurites of brains of patients with AD is compatible with autophagy impairment [7]. Further studies *in vivo* and *in vitro* demonstrated that both autophagy induction and autophagosome clearance may be altered in AD.

## Autophagy and Aβ

The amyloidogenic form of Aβ is generated from APP via sequential proteolytic cleavages performed by β- and γ-secretases. In AD, the production of Aβ exceeds its clearance, leading to Aβ accumulation and the formation of neurotoxic aggregates [6]. The latter might be caused, at least partially, by a progressive impairment of autophagy. Defects in autophagosome formation have been reported in brains of patients with AD and might be explained by reduced levels of BECLIN 1 and NBRF2, key regulators of autophagosome biogenesis. Genetic depletion of these factors in AD mouse models suppresses autophagy-mediated degradation of Aβ aggregates and accelerates cognitive impairment [8,9]. Accumulation of autophagosomes within patient dystrophic neurites suggests defective axonal retrograde transport. Studies in relevant mouse models demonstrate that interactions of Aβ oligomers with dynein motor protein hinder dynein recruitment to SNAPIN-loaded autophagosomes, thus impairing their trafficking towards lysosomes and reducing degradation of autophagy cargo [10]. Accordingly, dynein or SNAPIN overexpression in AD mice improved axonal transport, autophagosome maturation and Aβ clearance [10,11].

AD-related mutations in *PSEN1* and *PSEN2* not only cause pathogenic Aβ cleavage but also impact autophagy flux. PSEN1 maintains lysosomal acidification by targeting the v-ATPase V0a1 subunit to lysosomes, whereas PSEN2 controls RAB7 recruitment to autophagosomes by modulating ER Ca^2+^ homeostasis. Consequently, mutations in *PSEN1* and *PSEN2* impair lysosomal proteolysis and hinder autophagosome–lysosome fusion [12,13]. Environmental and genetic disease modifiers also contribute to AD by interfering with the autophagy–lysosomal pathway. For instance, elevated intracellular cholesterol levels in neurons exert a dual effect on autophagy and mitophagy. On the one hand, this stimulates auto/mitophagosome formation by exacerbating Aβ-induced mitochondrial oxidative stress, but, on the other hand, it alters the distribution of SNAREs and prompts the cytosolic aggregation of the mitophagy adaptor optineurin (OPTN), ultimately preventing cargo recognition and vesicle fusion necessary for the completion of auto/mitophagy [14,15]. Finally, components of the autophagic machinery display a protective role in AD via non-canonical pathways that use some components of the autophagy machinery. Murine studies show that LC3-associated phagocytosis and endocytosis are required for the recycling of Aβ receptors by microglia and defects in these processes contribute to Aβ accumulation, resulting in pervasive neurodegeneration, consistent with the human disease [16,17].

## Autophagy and tau

Tau is involved in microtubule assembly and stability, and it is modulated by several post-translational modifications. Among these, phosphorylation plays a central role in the formation of NFTs because hyperphosphorylation decreases tau association with microtubules, leading to protein misfolding and oligomerisation. Tau is also secreted, and its aggregates can spread from cell to cell to seed new aggregates in healthy cells [18]. Tau hyperacetylation by histone acetyltransferase p300 (p300)/CREB-binding protein (CBP) promotes its accumulation and causes cognitive deficits in transgenic mice [19]. p300 also mediates the acetylation of regulatory associated protein of mTOR, a component of mTORC1, which inhibits autophagy [20]. Hyperactivity of p300/CBP inhibits autophagy in tauopathy brains and promotes tau secretion in neurons. Enhancing autophagic flux prevents p300-mediated tau secretion, and inhibition of p300/CBP can reduce the spread of aggregated tau [21].

Pathological transformations of tau may also affect its interaction with the autophagic machinery. While normal and soluble forms of tau are targeted by the cargo receptor OPTN, the degradation of insoluble species via selective autophagy appears to be assisted by p62/SQSTM1. Interestingly, overexpression of p62/SQSTM1 reduces pathological tau and spreading in tauopathy mouse models [22]. Furthermore, slingshot homologue 1–mediated dephosphorylation of p62/SQSTM1 phospho-Ser403—a post-translational modification that enhances its activity and is decreased in AD—impairs its affinity for ubiquitinated substrates and tau clearance. Knockdown of slingshot homologue 1 decreases phospho-tau in PS19 mice brains [23].

Pathogenic tau may also amplify its toxic effects by inhibiting autophagy. Tau accumulation upregulates acidic leucine-rich nuclear phosphoprotein 32 family member A, a component of the histone acetyl transferase INHAT complex that masks histone acetylation. This inhibits the expression of increased sodium tolerance protein 1, which facilitates the assembly of the ESCRT-III complex required for autophagosome maturation [24]. Moreover, WT tau seeds are more efficiently targeted by autophagy than the P301L mutant counterparts in neurons. This may, in part, result from the degree of inhibition of histone deacetylase 6, involved in autophagosome–lysosome fusion, by the different seeds [25].

## Autophagy and inflammation

Microglia, the resident immune cells of the central nervous system, play a major role in regulating brain inflammation and have recently gained attention as key regulators of neurodegenerative processes. Coincidentally, autophagy is recognised as an essential modulator of inflammation. An example of the autophagic regulation of inflammation is the degradation of the NOD-, LRR- and pyrin domain-containing protein 3 (NLRP3) inflammasome, an intracellular complex responsible for the maturation of inflammatory cytokines interleukin-1β and interleukin-18 [26]. Beclin 1 insufficiency leads to an upregulation of NLRP3 in microglia, accompanied by elevated inflammatory cytokine expression [26]. Conversely, overexpression of TFEB promotes lysosomal function and decreases inflammatory protein expression in microglia from murine AD models [27]. These data suggest that the NLRP3 inflammasome is autophagy regulated and that autophagy deficiency directly causes inflammation. The autophagic machinery crosses paths with another key modulator of inflammation. Receptor-interacting serine/threonine-protein kinase 1 (RIPK1) regulates tumour necrosis factor (TNF)–induced cell death, balancing between apoptosis and necroptosis, that is between non-inflammatory and highly inflammatory cell death [28]. ULK1 phosphorylates RIPK1, thereby reducing TNF-induced cell death. Accordingly, ULK1 knockdown enhances TNF-induced necroptosis, providing further evidence of the anti-inflammatory role of autophagy [29]. Further cellular studies suggest that RIPK1 is a negative regulator of mTORC1, as its ablation enhances mTORC1 activity and lysosomal dysfunction [30]. In contrast, inhibition of RIPK1 in an AD mouse model reduced amyloid burden, the levels of inflammatory cytokines, and promoted microglial degradation of Aβ *in vitro* [31].

Finally, inflammatory cytokines can also impact autophagy. Interferon gamma administration promotes microglial clearance of Aβ in a mouse model of AD. Interferon gamma upregulates ATG5 and ATG7 expression, while simultaneously downregulating mTOR activity, indicating that the Aβ clearance might be autophagy-dependent [32].

## Autophagy-based therapeutic strategies for AD

Mounting evidence of a causal link between impaired autophagy and defective clearance of Aβ and tau has uncovered an exciting opportunity to develop autophagy-targeted therapy for the treatment of AD. As genetic manipulation inducing autophagy has been reviewed elsewhere [33], here, we will focus on the pharmacological approaches reported in recent years (Table 1). Small molecule enhancers of autophagy can be classified as mTOR-dependent or mTOR-independent. Classical allosteric mTOR inhibitors, including rapamycin and rapalogs, efficiently induce neuronal autophagic clearance of tau and Aβ and improve the cognitive function of several AD mouse models [33]. Similarly, treatment with ATP-competitive mTOR kinase inhibitors (OSI-027, AZD2014 and AZD8055) has been shown to persistently reduce tau levels in induced pluripotent stem cell-derived neurons from other tauopathies [34]. The development of compounds targeting upstream regulators of mTOR is another approach that can be used. Alborixin (an ionophore antibiotic) and nitazoxanide (an anti-parasitic drug) downregulate mTOR signalling via regulation of the PTEN/PI3K/AKT pathway, in the absence of toxicity. This results in increased autophagy-mediated clearance of Aβ in both neuronal and glial cells and improved learning and memory impairments of APP/PS1 transgenic mice [35,36]. Likewise, a tauopathy-homing nanoassembly containing PEGylated ceria nanoparticles modulates the mTOR–TFEB axis through AKT signalling. Treatment with this nanomaterial elicits autophagy-dependent tau proteolysis and ameliorates cognitive dysfunction in an AD rat model [37]. Finally, inhibition of p300-mediated acetylation by SMDC37892 also leads to a beneficial effect on tau turnover in human iPSC-derived excitatory neurons potentially due to mTORC1 inhibition caused by decreased raptor acetylation [20,21]. Although mTOR inhibition has shown promising effects, it may be important to consider the possibility raised by some studies suggesting that this strategy (and autophagy stimulation, in general) may only be beneficial if initiated early in the disease course [38].

**Table 1:** Molecules tested for amelioration of AD pathology through modulation of autophagy.

Molecule(s)	Target(s)	Mechanism of action	Effect in AD	Reference
OSI-027, AZD2014 and AZD8055	mTOR	ATP-competitive mTOR kinase inhibitors	Decrease in tau levels in iPSC-derived neurons from tauopathies	[34]
Alborixin and nitazoxanide	mTOR	Downregulation of mTOR signalling	Increased clearance of Aβ in neuronal and glial cells; improved learning and memory impairments of APP/PS1 transgenic mice	[35,36]
SMDC37892	mTOR	Modulation of p300-mediated acetylation	Tau turnover in human iPSC-derived excitatory neurons	[20,21]
Tauopathy homing nanoassembly	AKT/mTOR	mTOR–TFEB axis	Tau proteolysis; improvement of cognitive dysfunction in an AD rat model	[37]
Melatonin, metformin and crocetin	AMPK	Activation of AMPK	Aβ and tau clearance; improvement of cognitive decline in several tauopathies and APP/PS1 mice	[41, 42, 43]
Felodipine	AMPK	L-type Ca2+ channel antagonist	Reduces insoluble tau; ameliorates the morphologic abnormalities in zebrafish models of tauopathies	[45]
Sert	AMPK/mTOR	VDAC1-mediated mitochondrial transport of ATP	Tau degradation	[44]
Berberine	BECLIN 1/VPS34; CATHEPSIN D	Increases levels of BECLIN 1/VPS34; promotes maturation of lysosomal proteases cathepsin D	Tau clearance in 3 × Tg mice	[39]
ICCB-19 and Apt-1	BECLIN 1/VPS34 complex	Disrupt interaction of TRADD with TRAF2, cIAP1 or cIAP2, to increase K63-linked ubiquitination of Beclin1	Restores tau proteostasis in the PS19 mouse model	[40]
Curcumin analogue C1	TFEB	Activates TFEB nuclear translocation	Reduces APP, Aβ and tau aggregates in AD mouse models	[46,47]
Wy14643 and gemfibrozil	PPARα	Activation of PPARα	Decreases Aβ deposition and attenuates the cognitive deficits in APP/PS1ΔE9 mice	[48]
Thioperamide	H3R	CREB-mediated upregulation of TFEB/ATG7/LAMP1	Reduces Aβ levels in APP/PS1 mouse model	[49]
Desloratadine	5-HT2AR	SIRT1	Reduces Aβ levels in the APP/PS1 mouse model	[50]
CTEP	mGluR5	Modulation of the GSK3β–ZBTB16 autophagy pathway	Rescues Aβ pathology in male APPswe/PS1ΔE9 mice	[51]

See text for details.

The second category of compounds comprises mTOR-independent autophagy inducers. Berberine, an isoquinoline alkaloid, stimulates autophagy-mediated tau clearance in 3 × Tg AD mice by enhancing the level of BECLIN 1/VPS34 and promoting the maturation of lysosomal proteases CATHEPSIN D [39]. In addition, the small molecules ICCB-19 and Apt-1 enhance the autophagic turnover of tau by activating the BECLIN 1/VPS34 complex. Binding of these drugs to the adaptor protein TRADD displaces its interaction with TRAF2, cIAP1 or cIAP2, thus releasing these factors and allowing K63-linked ubiquitination of BECLIN 1. Treatment with Apt-1 also restored tau proteostasis in the PS19 mouse model [40].

Melatonin, metformin and crocetin increase both neuronal and microglial autophagy via activation of AMPK. Treatment with these compounds promotes Aβ and tau clearance and ameliorates cognitive decline in several tauopathies and APP/PS1 transgenic mouse models [41, 42, 43]. Sert, a serotonin selective reuptake inhibitor, modulates AMPK-mTOR-RPS6KB1 signalling by altering the VDAC1-mediated mitochondrial transport of ATP [44]. Similarly, the L-type Ca^2+^ channel antagonist felodipine diminished the neuronal levels of insoluble tau and ameliorates the morphologic abnormalities in zebrafish models of tauopathies [45].

TFEB is phosphorylated and inhibited by mTORC1; hence, most of the TFEB activators are mTOR inhibitors. Interestingly, the curcumin analogue C1 directly binds to and activates TFEB nuclear translocation without altering its phosphorylated state and acts independently of mTOR activity. This efficiently promotes autophagy flux and reduced APP, Aβ and tau aggregates in pre-pathological and symptomatic AD mouse models [46,47]. Similarly, drugs activating peroxisome proliferator-activated receptor alpha (Wy14643 and gemfibrozil), another transcription factor involved in the regulation of autophagy, decrease Aβ deposition and attenuate the cognitive deficits in APP/PS1ΔE9 mice [48]. Because of their wide expression in the brain and their role in modulating autophagy by sensing extracellular metabolites, G-protein–coupled receptors emerge as druggable targets in neurodegeneration. Chemical inhibition of H3R (thioperamide) and 5-HT2AR (desloratadine) promotes autophagy by CREB-mediated upregulation of TFEB/ATG7/LAMP1 (in neurons) and SIRT1 (in microglia), respectively, and significantly reduces Aβ levels in the APP/PS1 mouse model [49,50]. Interestingly, allosteric inhibition of mGluR5 by CTEP restarted autophagy and rescued Aβ pathology in male APPswe/PS1ΔE9 mice, but not in their female counterparts. This was due to the fact that the aberrant association of mGluR5 with Aβ and PrP^C^, which leads to the inactivation of the GSK3β–ZBTB16 autophagy pathway, occurs only in the male mouse brain [51]. In the context of gender-specific pathogenic events, the identification of the oestrogen receptor beta role in promoting autophagy flux by direct interaction with ATG7 might explain why low oestrogen levels during menopause correlate with an acceleration of AD onset [52]. Such insights will drive the future development of sex-tailored therapeutics.

## Concluding remarks

Pharmacological induction of autophagy reduces the levels of toxic aggregate-prone proteins and improves the clinical signs of the disease in multiple AD animal models. However, most of the models used so far are based on the genetic forms of AD; hence, they do not phenocopy the complex pathophysiology of most patients affected by the ‘sporadic’ disease. A future challenge will be to develop models incorporating non-genetic factors and the common comorbidities associated with AD and exploring the role of autophagy in this multifactorial context [53]. Upstream signalling regulators (mTOR, TFEB, AMPK) impacting autophagy have been exploited as druggable targets for improving the clearance of pathogenic aggregate-prone proteins. However, these factors act as central signalling hubs for many other vital functions in the cells; therefore, chronic pharmacological alteration of their activities may result in undesirable side effects. Considering that autophagy-based therapies are more efficient when administered in early stage of the disease and for long periods, the identification of more specific modulators with a better safety profile may be desirable [38]. Finally, recent publications shed light on the canonical and non-canonical functions of autophagy proteins in modulating the activity of microglia, which play a prominent role in the prodromal phase of the disease [54]. Enhancing autophagy in microglia counteracts inflammation and increases their phagocytic activity, which, in turn, prolongs the survival of neurons and delays disease progression. The introduction of measures targeting microglial autophagy may also hold promise for the future management of the disease.

## Conflict of interest statement

D.C.R. is a consultant for Aladdin Healthcare Technologies SE, Drishti Discoveries, PAQ Therapeutics and Nido Biosciences. None of the other authors have any potential competing interests.

## Financial support and sponsorship

This work was supported by UK Dementia Research Institute (funded by the MRC, Alzheimer's Research UK and the Alzheimer's Society), Roger de Spoelberch Foundation, Alzheimer's Research UK, The Tau Consortium, Cambridge Centre for Parkinson-Plus, National Institute for Health Research Cambridge Biomedical Research Centre, Merck Sharp & Dohme (D.C.R.) and European Union's Horizon 2020 research and innovation programme under the Marie Skłodowska-Curie grant agreement No 860035 (M.R.).

## References

[1] Feng Y., He D., Yao Z., Klionsky D.J. (2014). The machinery of macroautophagy. Cell Res.

[2] Levine B., Mizushima N., Virgin H.W. (2011). Autophagy in immunity and inflammation. Nature.

[3] Rubinsztein D.C., Mariño G., Kroemer G. (2011). Autophagy and aging. Cell.

[4] Dikic I., Elazar Z. (2018). Mechanism and medical implications of mammalian autophagy. Nat Rev Mol Cell Biol.

[5] Masters C.L., Bateman R., Blennow K., Rowe C.C., Sperling R.A., Cummings J.L. (2015). Alzheimer's disease. Nat Rev Dis Primer.

[6] Scheltens P., De Strooper B., Kivipelto M., Holstege H., Chételat G., Teunissen C.E., Cummings J., van der Flier W.M. (2021). Alzheimer's disease. Lancet Lond Engl.

[7] Nixon R.A., Wegiel J., Kumar A., Yu W.H., Peterhoff C., Cataldo A., Cuervo A.M. (2005). Extensive involvement of autophagy in Alzheimer disease: an immuno-electron microscopy study. J Neuropathol Exp Neurol.

[8] Pickford F., Masliah E., Britschgi M., Lucin K., Narasimhan R., Jaeger P.A., Small S., Spencer B., Rockenstein E., Levine B. (2008). The autophagy-related protein beclin 1 shows reduced expression in early Alzheimer disease and regulates amyloid beta accumulation in mice. J Clin Invest.

[9] Lachance V., Wang Q., Sweet E., Choi I., Cai C.-Z., Zhuang X.-X., Zhang Y., Jiang J.L., Blitzer R.D., Bozdagi-Gunal O. (2019). Autophagy protein NRBF2 has reduced expression in Alzheimer's brains and modulates memory and amyloid-beta homeostasis in mice. Mol Neurodegener.

[10] Tammineni P., Ye X., Feng T., Aikal D., Cai Q. (2017). Impaired retrograde transport of axonal autophagosomes contributes to autophagic stress in Alzheimer's disease neurons. eLife.

[11] Zhou F., Xiong X., Li S., Liang J., Zhang X., Tian M., Li X., Gao M., Tang L., Li Y. (2020). Enhanced autophagic retrograde axonal transport by dynein intermediate chain upregulation improves Aβ clearance and cognitive function in APP/PS1 double transgenic mice. Aging.

[12] Lee J.-H., Yu W.H., Kumar A., Lee S., Mohan P.S., Peterhoff C.M., Wolfe D.M., Martinez-Vicente M., Massey A.C., Sovak G. (2010). Lysosomal proteolysis and autophagy require presenilin 1 and are disrupted by Alzheimer-related PS1 mutations. Cell.

[13] Fedeli C., Filadi R., Rossi A., Mammucari C., Pizzo P. (2019). PSEN2 (presenilin 2) mutants linked to familial Alzheimer disease impair autophagy by altering Ca2+ homeostasis. Autophagy.

[14] Roca-Agujetas V., Barbero-Camps E., de Dios C., Podlesniy P., Abadin X., Morales A., Marí M., Trullàs R., Colell A. (2021). Cholesterol alters mitophagy by impairing optineurin recruitment and lysosomal clearance in Alzheimer's disease. Mol Neurodegener.

[15] Barbero-Camps E., Roca-Agujetas V., Bartolessis I., de Dios C., Fernández-Checa J.C., Marí M., Morales A., Hartmann T., Colell A. (2018). Cholesterol impairs autophagy-mediated clearance of amyloid beta while promoting its secretion. Autophagy.

[bib16] Heckmann B.L., Teubner B.J.W., Tummers B., Boada-Romero E., Harris L., Yang M., Guy C.S., Zakharenko S.S., Green D.R. (2019). LC3-Associated endocytosis facilitates β-amyloid clearance and mitigates neurodegeneration in murine Alzheimer's disease. Cell.

[17] Heckmann B.L., Teubner B.J.W., Boada-Romero E., Tummers B., Guy C., Fitzgerald P., Mayer U., Carding S., Zakharenko S.S., Wileman T. (2020). Noncanonical function of an autophagy protein prevents spontaneous Alzheimer's disease. Sci Adv.

[18] Brunello C.A., Merezhko M., Uronen R.-L., Huttunen H.J. (2020). Mechanisms of secretion and spreading of pathological tau protein. Cell Mol Life Sci CMLS.

[19] Min S.-W., Chen X., Tracy T.E., Li Y., Zhou Y., Wang C., Shirakawa K., Minami S.S., Defensor E., Mok S.A. (2015). Critical role of acetylation in tau-mediated neurodegeneration and cognitive deficits. Nat Med.

[20] Son S.M., Park S.J., Stamatakou E., Vicinanza M., Menzies F.M., Rubinsztein D.C. (2020). Leucine regulates autophagy via acetylation of the mTORC1 component raptor. Nat Commun.

[21] Chen X., Li Y., Wang C., Tang Y., Mok S.-A., Tsai R.M., Rojas J.C., Karydas A., Miller B.L., Boxer A.L. (2020). Promoting tau secretion and propagation by hyperactive p300/CBP via autophagy-lysosomal pathway in tauopathy. Mol Neurodegener.

[bib22] Xu Y., Zhang S., Zheng H. (2019). The cargo receptor SQSTM1 ameliorates neurofibrillary tangle pathology and spreading through selective targeting of pathological MAPT (microtubule associated protein tau). Autophagy.

[23] Fang C., Woo J.-A.A., Liu T., Zhao X., Cazzaro S., Yan Y., Matlack J., Kee T., LePochat P., Kang D.E. (2020). SSH1 impedes SQSTM1/p62 flux and MAPT/Tau clearance independent of CFL (cofilin) activation. Autophagy.

[24] Feng Q., Luo Y., Zhang X.-N., Yang X.-F., Hong X.-Y., Sun D.-S., Li X.-C., Hu Y., Li X.-G., Zhang J.-F. (2020). MAPT/Tau accumulation represses autophagy flux by disrupting IST1-regulated ESCRT-III complex formation: a vicious cycle in Alzheimer neurodegeneration. Autophagy.

[25] Tseng J.-H., Ajit A., Tabassum Z., Patel N., Tian X., Chen Y., Prevatte A.W., Ling K., Rigo F., Meeker R.B. (2021). Tau seeds are subject to aberrant modifications resulting in distinct signatures. Cell Rep.

[bib26] Houtman J., Freitag K., Gimber N., Schmoranzer J., Heppner F.L., Jendrach M. (2019). Beclin1-driven autophagy modulates the inflammatory response of microglia via NLRP 3. EMBO J.

[bib27] Zhou W., Xiao D., Zhao Y., Tan B., Long Z., Yu L., He G. (2021). Enhanced autolysosomal function ameliorates the inflammatory response mediated by the NLRP3 inflammasome in Alzheimer's disease. Front Aging Neurosci.

[28] Delanghe T., Dondelinger Y., Bertrand M.J.M. (2020). RIPK1 kinase-dependent death: a symphony of phosphorylation events. Trends Cell Biol.

[29] Wu W., Wang X., Berleth N., Deitersen J., Wallot-Hieke N., Böhler P., Schlütermann D., Stuhldreier F., Cox J., Schmitz K. (2020). The autophagy-initiating kinase ULK1 controls RIPK1-mediated cell death. Cell Rep.

[30] Najafov A., Luu H.S., Mookhtiar A.K., Mifflin L., Xia H.-G., Amin P.P., Ordureau A., Wang H., Yuan J. (2021). RIPK1 promotes energy sensing by the mTORC1 pathway. Mol Cell.

[31] Ofengeim D., Mazzitelli S., Ito Y., DeWitt J.P., Mifflin L., Zou C., Das S., Adiconis X., Chen H., Zhu H. (2017). RIPK1 mediates a disease-associated microglial response in Alzheimer's disease. Proc Natl Acad Sci U S A.

[bib32] He Z., Yang Y., Xing Z., Zuo Z., Wang R., Gu H., Qi F., Yao Z. (2020). Intraperitoneal injection of IFN-γ restores microglial autophagy, promotes amyloid-β clearance and improves cognition in APP/PS1 mice. Cell Death Dis.

[33] Djajadikerta A., Keshri S., Pavel M., Prestil R., Ryan L., Rubinsztein D.C. (2020). Autophagy induction as a therapeutic strategy for neurodegenerative diseases. J Mol Biol.

[34] Silva M.C., Nandi G.A., Tentarelli S., Gurrell I.K., Jamier T., Lucente D., Dickerson B.C., Brown D.G., Brandon N.J., Haggarty S.J. (2020). Prolonged tau clearance and stress vulnerability rescue by pharmacological activation of autophagy in tauopathy neurons. Nat Commun.

[35] Wani A., Gupta M., Ahmad M., Shah A.M., Ahsan A.U., Qazi P.H., Malik F., Singh G., Sharma P.R., Kaddoumi A. (2019). Alborixin clears amyloid-β by inducing autophagy through PTEN-mediated inhibition of the AKT pathway. Autophagy.

[36] Fan L., Qiu X.-X., Zhu Z.-Y., Lv J.-L., Lu J., Mao F., Zhu J., Wang J.-Y., Guan X.-W., Chen J. (2019). Nitazoxanide, an anti-parasitic drug, efficiently ameliorates learning and memory impairments in AD model mice. Acta Pharmacol Sin.

[37] Sun H., Zhong Y., Zhu X., Liao H., Lee J., Chen Y., Ma L., Ren J., Zhao M., Tu M. (2021). A tauopathy-homing and autophagy-activating nanoassembly for specific clearance of pathogenic tau in Alzheimer's disease. ACS Nano.

[38] Majumder S., Richardson A., Strong R., Oddo S. (2011). Inducing autophagy by rapamycin before, but not after, the formation of plaques and tangles ameliorates cognitive deficits. PloS One.

[39] Chen Y., Chen Y., Liang Y., Chen H., Ji X., Huang M. (2020). Berberine mitigates cognitive decline in an Alzheimer's Disease Mouse Model by targeting both tau hyperphosphorylation and autophagic clearance. Biomed Pharmacother.

[bib40] Xu D., Zhao H., Jin M., Zhu H., Shan B., Geng J., Dziedzic S.A., Amin P., Mifflin L., Naito M.G. (2020). Modulating TRADD to restore cellular homeostasis and inhibit apoptosis. Nature.

[41] Luengo E., Buendia I., Fernández-Mendívil C., Trigo-Alonso P., Negredo P., Michalska P., Hernández-García B., Sánchez-Ramos C., Bernal J.A., Ikezu T. (2019). Pharmacological doses of melatonin impede cognitive decline in tau-related Alzheimer models, once tauopathy is initiated, by restoring the autophagic flux. J Pineal Res.

[42] Wani A., Al Rihani S.B., Sharma A., Weadick B., Govindarajan R., Khan S.U., Sharma P.R., Dogra A., Nandi U., Reddy C.N. (2021). Crocetin promotes clearance of amyloid-β by inducing autophagy via the STK11/LKB1-mediated AMPK pathway. Autophagy.

[43] Chen Y., Zhao S., Fan Z., Li Z., Zhu Y., Shen T., Li K., Yan Y., Tian J., Liu Z. (2021). Metformin attenuates plaque-associated tau pathology and reduces amyloid-β burden in APP/PS1 mice. Alzheimer's Res Ther.

[44] Hwang H.-Y., Shim J.S., Kim D., Kwon H.J. (2020). Antidepressant drug sertraline modulates AMPK-MTOR signaling-mediated autophagy via targeting mitochondrial VDAC1 protein. Autophagy.

[bib45] Siddiqi F.H., Menzies F.M., Lopez A., Stamatakou E., Karabiyik C., Ureshino R., Ricketts T., Jimenez-Sanchez M., Esteban M.A., Lai L. (2019). Felodipine induces autophagy in mouse brains with pharmacokinetics amenable to repurposing. Nat Commun.

[46] Song J.-X., Malampati S., Zeng Y., Durairajan S.S.K., Yang C.-B., Tong B.C.-K., Iyaswamy A., Shang W.-B., Sreenivasmurthy S.G., Zhu Z. (2020). A small molecule transcription factor EB activator ameliorates beta-amyloid precursor protein and Tau pathology in Alzheimer's disease models. Aging Cell.

[47] Song J.-X., Sun Y.-R., Peluso I., Zeng Y., Yu X., Lu J.-H., Xu Z., Wang M.-Z., Liu L.-F., Huang Y.-Y. (2016). A novel curcumin analog binds to and activates TFEB in vitro and in vivo independent of MTOR inhibition. Autophagy.

[48] Luo R., Su L.-Y., Li G., Yang J., Liu Q., Yang L.-X., Zhang D.-F., Zhou H., Xu M., Fan Y. (2020). Activation of PPARA-mediated autophagy reduces Alzheimer disease-like pathology and cognitive decline in a murine model. Autophagy.

[49] Wang J., Liu B., Xu Y., Yang M., Wang C., Song M., Liu J., Wang W., You J., Sun F. (2021). Activation of CREB-mediated autophagy by thioperamide ameliorates β-amyloid pathology and cognition in Alzheimer's disease. Aging Cell.

[50] Lu J., Zhang C., Lv J., Zhu X., Jiang X., Lu W., Lu Y., Tang Z., Wang J., Shen X. (2021). Antiallergic drug desloratadine as a selective antagonist of 5HT2A receptor ameliorates pathology of Alzheimer's disease model mice by improving microglial dysfunction. Aging Cell.

[bib51] Abd-Elrahman K.S., Albaker A., de Souza J.M., Ribeiro F.M., Schlossmacher M.G., Tiberi M., Hamilton A., Ferguson S.S.G. (2020). Aβ oligomers induce pathophysiological mGluR5 signaling in Alzheimer's disease model mice in a sex-selective manner. Sci Signal.

[52] Wei Y., Zhou J., Wu J., Huang J. (2019). ERβ promotes Aβ degradation via the modulation of autophagy. Cell Death Dis.

[53] Dawson T.M., Golde T.E., Lagier-Tourenne C. (2018). Animal models of neurodegenerative diseases. Nat Neurosci.

[54] Plaza-Zabala A., Sierra-Torre V., Sierra A., Autophagy, Microglia (2017). Novel partners in neurodegeneration and aging. Int J Mol Sci.

